# VEGF-mTOR signaling links obesity and endometrial cancer

**DOI:** 10.18632/oncoscience.430

**Published:** 2018-06-27

**Authors:** Subhransu S. Sahoo, Pradeep S. Tanwar

**Affiliations:** Gynecology Oncology Group, University of Newcastle, Callaghan, NSW 2308, Australia

**Keywords:** fat, BMI, cancer, gynaecological cancers, uterus

Over past few decades, the incidence of endometrial cancer (cancer of the uterine lining) continues to grow at a faster rate among all the gynecological cancer types. Out of several risk factors (genetic predisposition, family history, hormone replacement therapy, early menarche, and late menopause), lifestyle factors such as high-fat diet and overweight has a significant contribution towards this unfortunate trend. A recent statistic shows that more than half of endometrial cancers (57%) are due to obesity [1]. In addition, epidemiological evidence from both case-control and cohort studies show a linear rise in the risk of endometrial cancer with increasing BMI (body mass index). As the global obesity epidemic has shown no signs of abating, particularly in the western countries, it is speculated an endometrial cancer tsunami is coming!

In case of obesity, the number of adipose progenitor cells increases, which synthesize surplus adipocytes. These hypertrophied adipocytes play a relevant role in endometrial cancer patients via several pathways such as insulin resistance, leptin resistance, secretion of adipokines, and *in situ* synthesis of estrogen [2]. Of note, insulin resistance has been observed in case of type 2 diabetes patients, as well as excess estrogen in women, also potentiates breast and ovarian cancer. Thus, at present, the biological mechanism clearly linking the strongest association of overweight or obesity to endometrial cancer is poorly understood. The molecular mechanisms by which cancer cell-adipocyte interactions promote malignant growth vary among tumor types. Although clinical and epidemiological data strongly support endometrial cancer risk in obese women, these concepts have yet to be characterized at the molecular level to be effectively translated into the clinical impact.

We recently demonstrated the existence of a regulatory axis between visceral adipose tissue (VAT) and endometrial cancer cells [3]. Vascular endothelial growth factor (VEGF) was suggested (Figure 1) as the VAT-released growth factor, which induced mTOR signaling in endometrial cancer cells and proliferation of these cells. These findings were demonstrated *in vitro* and in a xenograft model, using human endometrial cancer cell lines and primary adipose tissue samples. We also described that the high VEGF release leads to up-regulation of the phosphorylated form of AKT and S6 protein in xenograft of endometrial cancer cells when exposed to the VAT, further supporting the cooperation of VEGF and mTOR signaling in tumor progression [3].

**Figure 1:**
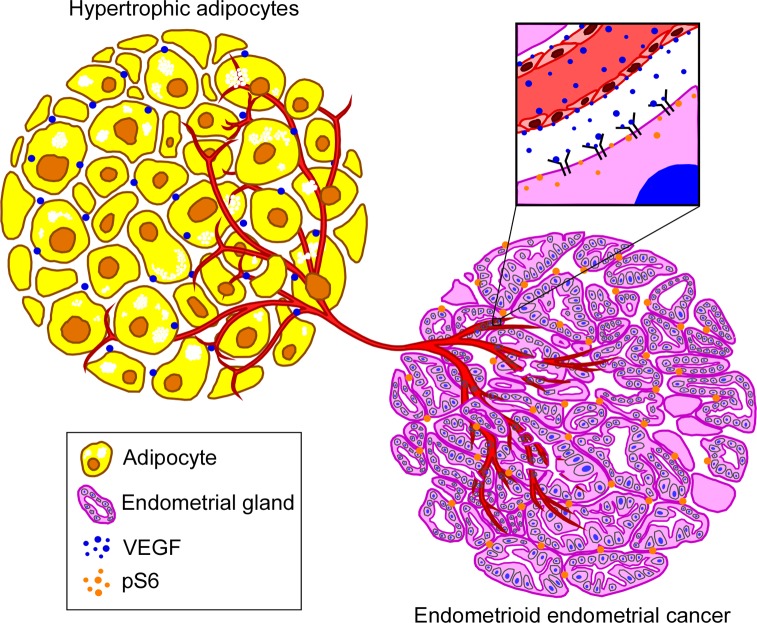
Visceral adiposity drives endometrial cancer growth Vascular endothelial growth factor (VEGF) secreted by adipocytes promotes angiogenesis in the uterus of obese mice or women. Feedforward effects of VEGF on pVEGFR2 and subsequent increase in pS6 expression in the uterine glands drive endometrial cancer initiation and growth.

More than 80% of endometrial cancers are adenocarcinomas (Type I) or cancers of the cells that form glands in the endometrium [4]. We, therefore, reconsidered whether adipocyte-derived VEGF stimulates mTOR signaling in endometrial glands. Remarkably, by using a hyperphagic obese mouse model, we discovered that with age as the mouse gains body weight due to abdominal fat deposition, the number of endometrial glands in the uterus increased as compared to its non-obese littermate. The obese mice uterus exhibited elevated VEGF and pVEGFR2 with upregulated pS6 expression in endometrial glands supporting a direct effect of visceral adiposity towards endometrial adenocarcinoma [3]. This is further evidenced in high BMI human endometrial cancer patients where a positive correlation was found between VEGF or pS6 expression towards BMI of patients.

Majority of type I endometrial cancer patients have genetic aberrations in the members of the PI3K-mTOR pathway [4]. Recently our group has shown upregulation of mTOR signaling in postmenopausal hyperplastic endometrium compared to postmenopausal normal controls [5]. Thus, in obese women, it is highly plausible that the excess VEGF secreted from VAT provides an extra fuel to activate mTOR that can trigger endometrial hyperplasia or cancer at an early stage or among young women. Furthermore, this report raises a number of other interesting questions. (1) How important is adipocyte-derived VEGF-mTOR signaling in normal physiology? (2) Previously we have reported that pharmacological suppression of the mTOR pathway using rapamycin treatment significantly reduces endometrial hyperplasia in aged mice [5]. Metformin is another potential chemopreventive medication, which increases insulin sensitivity as well as activates the AMPK pathway and stimulates a tumor suppressive gene known as *LKB1*, thereby counteracts the PI3K/AKT/mTOR signaling pathway [6]. Thus, whether the use of a combination of rapamycin and metformin could effectively suppress the hyperactive mTOR signaling in obese women uterus, potentially lowering the risk of endometrial cancer. (3) Are the blood vessels primary link between VAT and uterus? What if the communication between VAT and female reproductive tract would be interrupted by use of an anti-angiogenic drug such as bevacizumab [7], would there be a reduced effect of VEGF on uterus? Moving forward, in addition to a healthy lifestyle (low-calorie food and exercise), our recent study opens a new avenue to exploit the analysis of anti-mTOR and anti-VEGF therapy in obese women to prevent or treat endometrial cancer.

## References

[1] Onstad MA (2016). J Clin Oncol.

[2] Khandekar MJ (2011). Nat Rev Cancer.

[3] Sahoo SS (2018). Mol Cancer Res.

[4] Morice P (2016). Lancet.

[5] Bajwa P (2017). Oncotarget.

[6] Taubes G. (2012). Science.

[7] Cao Y (2011). Sci Transl Med.

